# Exploring the antioxidant potential of endophytic fungi: a review on methods for extraction and quantification of total antioxidant capacity (TAC)

**DOI:** 10.1007/s13205-024-03970-3

**Published:** 2024-04-05

**Authors:** Rita Onyekachukwu Asomadu, Timothy Prince Chidike Ezeorba, Tobechukwu Christian Ezike, Jude Obiorah Uzoechina

**Affiliations:** 1 Department of Biochemistry, Faculty of Biological Sciences, University of Nigeria, Enugu, 410001 Nigeria; 2 Department of Genetics and Biotechnology, Faculty of Biological Sciences, University of Nigeria, Enugu, 410001 Nigeria; 3 Department of Environmental Health and Risk Management, College of Life and Environmental Sciences, University of Birmingham, Edgbaston, B17 2TT UK

**Keywords:** Oxidative stress, Endophyte solvent extracts and secondary metabolites, Total Phenolic content (TPC), Total flavonoid content (TFC), DPPH, FRAP, ABTS, ORAC

## Abstract

Endophytic fungi have emerged as a significant source of natural products with remarkable bioactivities. Recent research has identified numerous antioxidant molecules among the secondary metabolites of endophytic fungi. These organisms, whether unicellular or micro-multicellular, offer the potential for genetic manipulation to enhance the production of these valuable antioxidant compounds, which hold promise for promoting health, vitality, and various biotechnological applications. In this study, we provide a critical review of methods for extracting, purifying, characterizing, and estimating the total antioxidant capacity (TAC) of endophytic fungi metabolites. While many endophytes produce metabolites similar to those found in plants with established symbiotic associations, we also highlight the existence of novel metabolites with potential scientific interest. Additionally, we discuss how advancements in nanotechnology have opened new avenues for exploring nanoformulations of endophytic metabolites in future studies, offering opportunities for diverse biological and industrial applications.

## Introduction

A growing body of evidence has shown that reactive oxygen species (ROS) are constantly generated even during normal cellular activities and must be carefully regulated in living organisms. While ROS play a role in destroying parasitic invaders, uncontrolled ROS levels can lead to systemic oxidative stress, causing damage to biological macromolecules and contributing to various debilitating conditions such as cancer, neurodegenerative disorders, diabetes, and aging (Sharifi-Rad et al. 2020; Okeke et al. 2021; Okagu et al. 2022; Chukwuma et al. 2023a, b). Additionally, ROS and free radicals can foster lipid peroxidation, the destruction of innate antioxidant enzymes, the oxidation of other proteins and enzymes, and other metabolic disturbances (He et al. 2017).

Humans have traditionally relied on diverse medicinal plants for their radical scavenging ability and antioxidant capacity, which are attributed to their secreted metabolites and phytochemicals such as phenolics, alkaloids, amines, and terpenoids (Tungmunnithum et al. 2018). However, due to competing needs for other plant resources, as well as challenges posed by overexploitation and urbanization, the availability of medicinal plants and their resources for combating oxidative stress may be limited (Chen et al. 2016; Chukwuma et al. 2022; Enechi et al. 2022; Omeje et al. 2023). Therefore, there is a need for alternative sources of antioxidant molecules, and endophytic fungi (EFI) represent promising candidates (Wen et al. 2022).

Endophytic fungi (EFI) are ubiquitous endophytes that primarily inhabit the aerial tissues of various plants without causing noticeable harm or infectious symptoms (Chauhan et al. 2019). In symbiosis with the host plant, EFI provide defense against pathogens and herbivores while obtaining nutrients and protection. There are over a million diverse EFI species found in various plants, although it is important to note that EFI species are not specific to individual plants (Huang et al. 2007; Manganyi and Ateba 2020). EFI present an underexplored source of structurally unique natural products with diverse activities for medicinal and industrial purposes. While contemporary research has primarily focused on understanding the potential bioactivities of EFI from medicinal and ethnopharmacological plants, the potential of EFI from non-medicinal plants for industrial applications has been largely overlooked (Caruso et al. 2020).

Antioxidant activity differs from antioxidant capacity, with the former being a kinetic-based estimation of the rate constant of redox reactions or radical scavenging activities per time. Antioxidant capacity, on the other hand, measures the efficiency of counteracting the oxidative degradation of biological macromolecules by different antioxidants (Bunaciu et al. 2016). Total antioxidant capacity (TAC) estimates the total or resultant antioxidant capacity contributed synergistically or antagonistically by individual components of a complex mixture or a biological system (Flieger et al. 2021). Obtaining the TAC of endophytic metabolites may not be straightforward, as different antioxidant components exhibit different mechanisms of action. While some act as free radical scavengers, others may prevent ROS formation entirely. Additionally, certain metabolites may induce signaling pathways to repair oxidative damage (Wen et al. 2022). The diverse routes of antioxidant action pose challenges in accurately estimating the TAC of a biological system (Verma et al. 2022). The difficulty is further compounded when estimating TAC in vivo due to contributions from natural antioxidant enzymes and individual variations in metabolism (Fadiji and Babalola 2020). As a result, there are no standard reference values for the normal TAC ranges of different in vivo models (Shahidi and Zhong 2015).

Conversely, in vitro estimation of TAC has been more reliable, although different assays often yield different results. Therefore, it is essential to understand the principles and nature of the antioxidant function estimated by any selected in vitro TAC model (Manganyi and Ateba 2020). While some models estimate primary antioxidants through their ability to scavenge free radicals via hydrogen transfer or electron transfer, others estimate secondary antioxidants through their chelating ability of transition metals or inhibition activities of Fenton reactions or Lewis acid–base neutralization (Flieger et al. 2021).

**Fig. 1:**
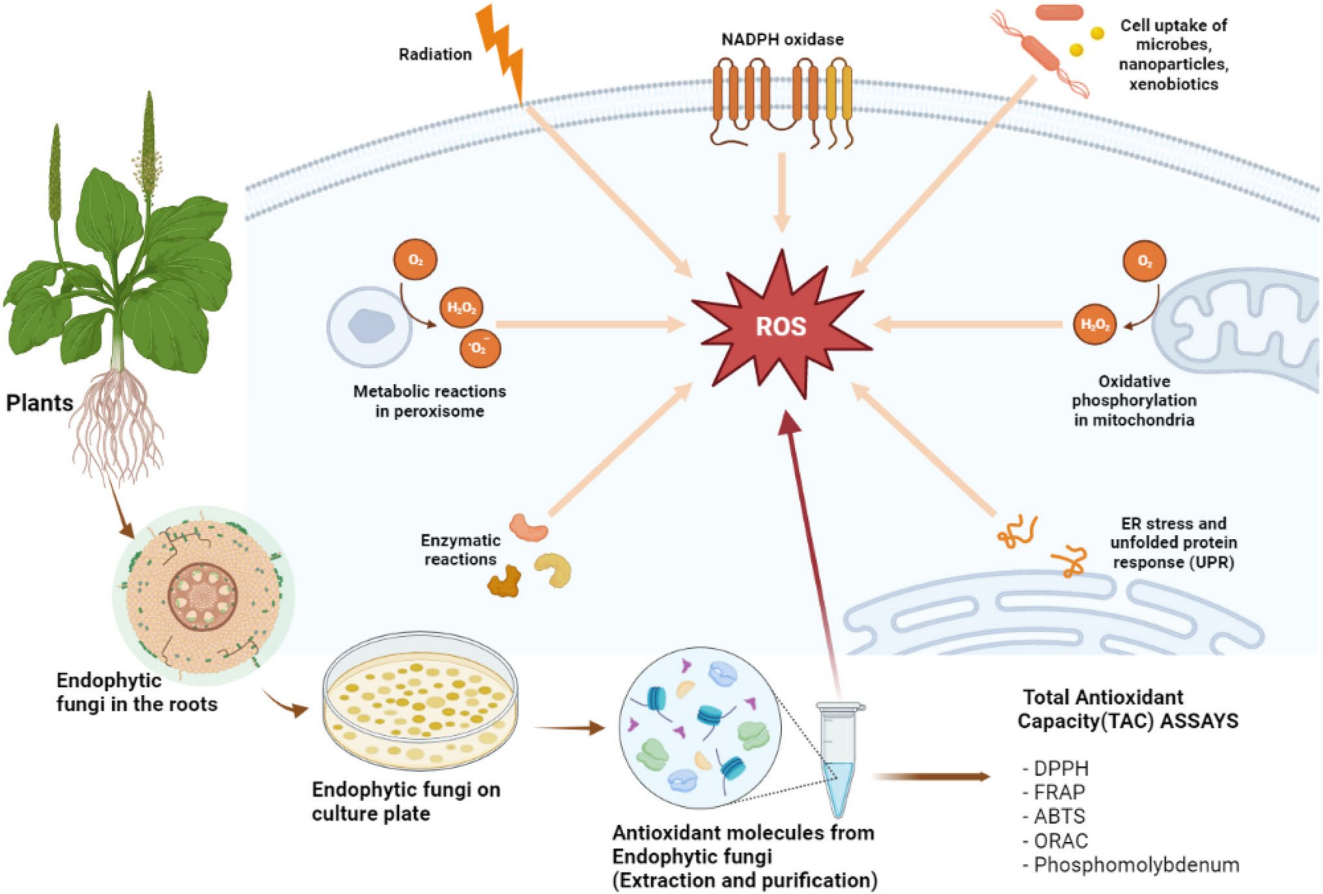
An illustrative description of the processes for extracting and purifying endophytic fungi metabolites from plants and the importance of its antioxidant molecules on cellular homeostasis and popular methods for estimating their total antioxidant capacity (TAC), including DPPH, FRAP, ABTS, and ORAC

This study provides an updated review of the cultivation, extraction, and purification of antioxidant metabolites from endophytic fungi, highlighting several optimized methodologies for contemporary research that yield valid and verifiable evidence. Additionally, this study delves into various in vitro models for Total Antioxidant Capacity (TAC) estimation of endophytic fungi, examining both competitive and non-competitive models (see Fig. 1). The present limitations confronting studies on endophytic fungi and their metabolites are discussed, and potential avenues for future research are suggested.

## Methodology

The methodology employed in this study follows a traditional literature review approach. A systematic search was conducted in databases including Scopus, PubMed, and ScienceDirect using specific keywords such as "Endophytes," "Endophytic fungi," "Antioxidant," "Total antioxidant capacity," "radical scavenging," "extraction," "isolation," "purification," "FRAP," "DPPH," "ABTS," and "nanoparticles." Boolean connectors such as AND or OR were utilized to refine the search and ensure relevance. The search was limited to studies published after 2015, and only articles written in English were considered. Studies that did not focus on Endophytic fungi metabolites were excluded during the screening process.

## Nature and sources of endophytic fungi and their metabolites

Endophytes, a diverse group of microorganisms residing asymptomatically within various plant tissues (Grabka et al. 2022; Tsivileva et al. 2022), thrive in terrestrial, mangrove, and marine habitats (Mishra et al. 2019). Plants are reservoirs of numerous bioactive metabolites with diverse pharmacological properties. The exploration of endophytes within these plants yields similar compounds, facilitated by a long evolutionary relationship and genetic transfer. This not only enables large-scale metabolite production but also aids in biodiversity preservation (Singh et al. 2016). Endophytic fungi, alongside endophyte bacteria or actinomycetes, are esteemed sources of natural chemical structures possessing antioxidant activity (Toghueo and Boyom 2019; Tsivileva et al. 2022), in addition to producing novel compounds with significant biological attributes (Toghueo and Boyom 2019).

The validation of bioactive secondary metabolites' production by numerous fungi, emphasized by Strobel and Daisy (2003), underscores the immense potential of endophytes as repositories of secondary metabolites. Various organs of known higher plants serve as potential habitats for these life forms (Table 1), yielding a wide array of bioactive metabolites with therapeutic potential for various diseases (Hardoim et al. 2015; Ejaz et al. 2020). Endophytic fungi stand out as primary producers of diverse metabolites with potent biological activities encompassing antioxidant, antibiotic, antidiabetic, anticancer, and immunosuppressive properties (Gao et al. 2018). Their coexistence with host plants, predominantly higher plants, harbors diverse microorganisms capable of synthesizing novel metabolites and compounds (Deshmukh et al. 2018).

**Table 1:** Selected endophytic fungi and their plant sources

Endophytic fungi	Host plant	Plant part	References
*Penicillium oxalicum* *Thielavia subthermophila*	*Gymnema sylvestre* *Hypericum perforatum*	Whole plant	(Paramanantham et al. 2019)
*Corynespora cassiicola, Scopulariopsis brevicaulis,* *Guignardia mangiferae,* *Cladosporium sp. Cladosporioides sp.* *Trichoderma koningii,* *Alternaria alternate,* *Fusarium sp.,* *Aspergillus sp.,* *Penicillium sp.*	*Glycine max* *Zea mays*	Whole plant	(Kumar et al. 2019)
*Aspergillus allahabadii* *Aspergillus terreus* *Aspergillus tubingensis* *Aspergillus tamarii* *Aspergillus micronesiensis* *Aspergillus fumigatus* *Aspergillus flocculus* *Aspergillus flavipes* *Aspergillus flavus* *Aspergillus calidoustus* *Aspergillus capensis*	*Cinnamomum subavenium* *Carthamus lanatus* *Lycium ruthenicum* *Ficus carica* *Phyllanthus glaucus* *Erythrophleum fordii* *Markhamia platycalyx* *Suaeda glauca* *Cephalotaxus fortune* *Acanthospermum australe* *Brassica napus L*	Root, stem, and leaves	(El-Hawary et al. 2020) (De Carvalho et al. 2016; Qin et al. 2019a, b)
*Alternaria alternata, Cladosporium herbarum, Epicoccum nigrum,* *Cryptococcus sp.,* *Rhodotorula rubra,* *Penicillium sp., and Fusarium graminearum*	*Wheat*	Whole plant	(Souza and Santos 2017)
*Alternaria tenuissima*	*Erythrophleum fordii,* *Acacia magnum*	Bark and leaves	(Eram et al. 2018)
*Colletotrichum* *Cladosporium* *Phomopsis*	*Artemisia mongolica* *Artemisia annua* *Baccharis dracunculifolia* *Cichorium intybus* *Achillea millefolium* *Anthemis segetali* *Viguiera arenaria* *Notobasis syriaca*	Stem *Stem, leaf, and root* *inflorescence*	(Caruso et al. 2020)
*Guignardia sp* *Pezicula sp.* *Trichothecium sp.* *Berkleasmium sp.* *Phialophora mustea*	*Euphorbia sieboldiana* *Forsythia viridissima* *Phyllanthus amarus* *Dioscorea zingiberensis* *Crocus sativus*	Stem and leaves	(Deshmukh et al. 2018)
*Penicillium sp.* *Chaetomium sp.* *Sporormiella minimoides* *Trichoderma spirale* *Colletotrichum sp.*	*Alibertia macrophylla* *Hintonia latiflora* *Morinda officinalis* *Rubia pondantha*	Leaves and stem	(Cruz et al. 2020)
*Phoma multirostrata EA-12* *Penicillium brefeldianum* *Phomopsis glabrae* *Aspergillus tamarii* *Epicoccum nigrum*	*Eupatorium adenophorum* *Pinellia ternata* *Pongamia pinnata* (L.) *Ficus carica* *Pinellia ternate* *Mentha suaveolens*	Fresh leaves, Rhizome, and Roots	(Li et al. 2018)

These secondary metabolites are often produced by organisms in response to external stimuli such as foreign infections or changes in nutrition. Endophytes, in particular, respond to various environmental changes and stimuli, including nutritional shifts and attacks by foreign pathogens, by producing these secondary metabolites (Ejaz et al. 2020). Consequently, endophytes influence the physiological activities of host plants, including resistance to diseases, nematodes, insects, and stress enhancement.

Over the past 15 years, research has unveiled a diverse array of metabolites across different chemical classes, including alkaloids, terpenes, isocoumarins, xanthones, cytochalasins, polyketides, phenols, phenolic acids, steroids, quinones, aliphatic acids, and chlorinated compounds (Deshmukh et al. 2018; Cruz et al. 2020; Wen et al. 2022).

### (a) Alkaloids

Alkaloids constitute a vast group of nitrogenous organic compounds with low molecular weight derived from amino acids, frequently encountered in various living organisms, such as bacteria, fungi, plants, and animals (Moreira et al. 2018). Examples of alkaloids isolated from different species of endophytic fungi include cytochalasin D, quinine, brevianamide F, 11-bromoroquefortine, phomopsisin A–C, diaporthichalasins D–H, and Cytochrysins A–C (Cafêu et al. 2005; Da Silva et al. 2014; Maehara et al. 2016; Yang et al. 2019; Wen et al. 2022).

### (b) Terpenes

Terpenes represent the most assorted and diverse group of compounds occurring naturally, primarily found in plants (Cox-Georgian et al. 2019). A terpene molecule is derived from at least two isoprene units, typically containing 10 carbon atoms. Monoterpenes, sesquiterpenes, diterpenes, and triterpenes are generally composed of two, three, four, or six isoprene units (Rocha-Santos and Duarte 2019). Larger classes such as squalene and sterols can be found in animals (Cox-Georgian et al. 2019). Some terpenes obtained from various endophytic fungi include koninginols A and B, guignardone I, colletotrichine A and B, stelliophaerols A and B, lithocarin B and C, and tenellone H (Cruz et al. 2020).

### (c) Quinones

Quinones represent a class of naturally occurring compounds and synthetic molecules with several beneficial effects. However, they also possess toxicological effects because they exist in air pollutants as photoproducts (El-Najjar et al. 2011). The various forms in which quinones exist in nature are benzoquinones, anthraquinones, polycyclic quinones, and naphthoquinones. Examples of quinones isolated from different endophytic fungi include 1,3-dihydroxy-4–8-methoxy-9H-xanthen-9-one, a new quinone obtained from the endophytic fungus *Phomopsis sp*. isolated from the rhizome of Paris polyphylla var. from Yunnan, province China (Wen et al. 2022). Others include 6-epi-stemphytriol, dihydroalterperylenol, alterperylenol, altertoxin II, and stemphyperylenol, all extracted from the endophytic fungus *Alternaria sp.* obtained from *Morinda officinalis* (Cruz et al. 2020).

### (d) Phenolic compounds

Phenolic compounds are bioactive substances or compounds with one or more aromatic rings whose structures contain hydroxyl groups. They include simple phenols, polyphenols, and flavonoids (Lin et al. 2016). Some phenolic compounds extracted from various endophytic fungi are orcinol, thielavins A, J, and K, cytosporaphenone A, and resorcinol (Rivera-Chávez et al. 2013; Cruz et al. 2020).

### (e) Polyketides

Polyketides are structurally diverse natural products and biologically active secondary metabolites synthesized by plants, bacteria, and endophytic fungi (Wang et al. 2020). Phomopsiketones A-C, (10S)-10-O-β-D-4’-methoxy-mannopyranosyl-diaporthin, and clearanol H were isolated from the endophytic fungus *Phomopsis sp*. obtained from stems of *Isodon eriocalyx var. laxiflora* (Tang et al. 2017). Colletopeptide A–D were extracted from *Colletotrichum sp*., an endophytic fungus isolated from *Rubia pondantha* (Feng et al. 2019). Mycoleptones A and B and austidiol were all extracted from *Mycoleptodiscus indicus* and endophytic fungi isolated from *Borreria verticillata* (Feng et al. 2019).

### (f) Steroids

Steroids are a group of biologically active secondary metabolites with diverse structures mainly derived from plants, animals, and fungi. All steroids have the basic perhydro-1,2-cyclopentenophenanthrene skeleton; however, introducing various functional groups results in slight variations leading to the various classes of steroids (Sultan and Raza 2015). Aspergilolide is a steroid lactone isolated from *Aspergillus sp.,* an endophytic fungus obtained from *Paeonia ostii* (Zhang et al. 2019). Phomopsterones are ergostane-type steroids obtained from the plant-derived endophytic fungus, *Phomopsis sp*. (Hu et al. 2017). Also, three known steroids, calvasterols A and B and ganodermaside D, were extracted from the culture broth of *Phomopsis sp*. and endophytic fungi isolated from *Aconitum carmichaelii* (Wu et al. 2013).

## Extraction, purification, and characterization of endophytic fungi metabolites

The extraction of bioactive metabolites from endophytic fungi stands as a pivotal step before their characterization and utilization. Initially, pure cultures of endophytes are cultivated in a defined liquid medium, and subsequently, their metabolites are extracted using suitable solvents or combinations thereof, based on the solubility of the target metabolites. A commonly employed method involves liquid–liquid extraction, where the organism is cultured in a liquid medium along with the organic solvent(s). Various solvents, including methanol, ethanol, ethyl acetate, dichloromethane (DCM), and hexane, have shown rapid extraction of bioactive metabolites from endophytic fungi (Madhusudhan et al. 2015).

For instance, bioactive metabolites from the endophyte *Pestalotiopsis neglecta BAB-5510* were extracted using an equimolar concentration of ethyl acetate and methanol. The resulting crude extract containing the active fraction was then subjected to drying in a rotary vacuum evaporator (Sharma et al. 2016). Similarly, in another study, a fungal strain in a liquid medium was incubated at room temperature for 6 weeks with ethyl acetate. The resulting crude mixture was vacuum filtered and then dried using a rotary evaporator (Ibrahim et al. 2021). Additionally, secondary metabolites from two *Cladosporium species* were concurrently extracted using ethyl acetate to yield a crude metabolite extract (Wats et al. 2021).

Furthermore, ethyl acetate was exclusively employed for the extraction of metabolites from endophytic fungi isolated from *Salvia abrotanoides*. The resulting ethyl acetate mycelia mixture was mechanically disrupted using glass beads for enhanced cell wall lysis. Subsequently, the homogenate was centrifuged, and the supernatant was utilized for further investigations (Teimoori-Boghsani et al. 2020). Conversely, Kumari et al. (2021) conducted extractions of secondary metabolites from the endophytic fungus *Penicillium citrinum* using ethyl acetate repeatedly for better extraction efficiency. Conversely, methanol alone was employed to extract bioactive metabolites from the endophytic fungus *Curvularia sp. T12,* resulting in a brown crude extract after concentration and drying (Kaaniche et al. 2018).

The extraction processes yield crude metabolites, which necessitate further purification to separate into component fractions for subsequent characterization. The isolation of pure metabolites is imperative for structural elucidation and identification. Various chromatographic methods, including column chromatography and high-performance liquid chromatography (HPLC), have been employed for purifying endophytic fungi metabolites (Kaaniche et al. 2018; Kumari et al. 2021). For example, a crude metabolite extract from the endophytic fungus *Penicillium citrinum* was fractionated into 11 bands using thin-layer chromatography, with a mobile phase comprising a mixture of chloroform, methanol, and ethyl acetate, and a stationary phase of silica gel (Kumari et al. 2021). Similarly, the crude metabolite extract from endophytic fungi isolated from *Ocimum basilicum* was fractionated into two white solid fractions (AH-1 and AH-2) using silica gel column chromatography, followed by recrystallization for further purification (Haque et al. 2005). Kaaniche et al. (2018) employed a two-step purification method involving silica gel column chromatography followed by gel filtration using Sephadex LH-20 to purify metabolites from the endophytic fungus *Curvularia sp. T12.*

Following the extraction and purification of metabolites from fungal cultures, the bioactive compounds present need to be identified and characterized. Analytical approaches for identifying and characterizing bioactive metabolites have advanced from simple to more sophisticated yet sensitive and rapid methods, resulting in less time-consuming and tedious secondary metabolite characterization with reproducible outcomes. Techniques such as gas chromatography (GC), nuclear magnetic resonance (NMR), and mass spectrometry (MS) are commonly employed for characterizing recovered active fractions (Madhusudhan et al. 2015). Mass spectrometry, for instance, is used to determine the mass of pure substances (MS).

Moreover, chromatography equipment, such as gas or liquid chromatography (GC or LC), is often coupled with a mass spectrometer. When an LC system is linked to an electrospray ionization (ESI) MS system, the sample can be dissolved, separated, transported, and ionized before entering the mass analyzer. For instance, taxol, a bioactive metabolite, was purified and quantified from the culture extract of the endophytic fungus taxus cuspidate using HPLC, and its structure was elucidated using LC–MS and 1H NMR spectroscopic analyses (Kumaran et al. 2010). Teimoori-Boghsani et al. (2020) isolated a bioactive compound, cryptotanshinone, from the endophytic fungi of native *Salvia abrotanoides* plants using high-resolution MS. Additionally, vinblastine and vincristine were extracted from the fungal endophyte *Fusarium oxysporum* and characterized using electrospray ionization mass spectrometry (ESI–MS) and tandem mass spectrometry (MS–MS) for molecular weight determination, followed by 1H NMR analysis for structural elucidation (Kumar et al. 2013).

Furthermore, the characterization of a purified fraction of bioactive metabolites from the endophytic fungus *Penicillium citrinum* of *Azadirachta indica* involved a combination of TLC, GC–MS, 1H NMR, and 13C NMR analyses. One of the identified bioactive compounds, milbemycin, exhibited antimicrobial activity against human pathogenic bacteria and fungi (Kumari et al. 2021). Given that endophytic fungi metabolite extracts comprise complex mixtures of several compounds, appropriate hyphenated techniques have proven effective for their separation and characterization. Techniques such as LC-PDA and LC–MS are commonly employed for the analysis of a wide range of endophytic metabolites, from small non-polar compounds to ample polar constituents. Moreover, LC-NMR and multiple-hyphenated techniques like LC-PDA-NMR-MS have gained acceptance as well (Madhusudhan et al. 2015).

## Methods for estimating the total antioxidant capacity (TAC), total phenolic content (TPC), and total flavonoid content (TFC) of endophytic fungi metabolites

Endophytic fungi derived from medicinal plants represent promising sources of antioxidants (Khiralla et al. 2015). Total Antioxidant Capacity (TAC) estimation provides a global assessment of a sample's ability to neutralize, scavenge, or mop up free radicals or reactive oxygen species (ROS). TAC offers insights into the antioxidant potency of an extract or sample without delving into the constituent antioxidants. Various methods have been employed to determine the total antioxidant activity, with results quantified by comparing them with the relative values of standard antioxidants such as trolox or ascorbic acid (Wen et al. 2022).

Methods for estimating TAC include the phosphomolybdenum method, DPPH assay, FRAP assay, and ORAC assay. While these methods may vary in principles, methodology, and sensitivity to different antioxidants, evidence from several studies suggests that methodologies used for plant metabolites are often analogous to those applied to endophytic fungi (Gupta et al. 2020). Furthermore, although plants and endophytes may produce similar metabolites, several studies have reported the presence of novel metabolites with unique MS/MS ions and remarkable antioxidant capacity in endophytic extracts. This has, therefore, paved the way for evaluating the structural properties of such metabolites (Wen et al. 2022).

Contemporary research has embraced methodologies that specifically assess antioxidant effects based on the Total Phenolic Content (TPC) or the Total Flavonoid Content (TFC). Total phenolic content expresses antioxidant capacity based on the cumulative components of flavonoids, phenolic acids, and other polyphenols (Gupta et al. 2020). TPC is commonly assayed using the Folin–Ciocalteu method, wherein Folin–Ciocalteu reagents, in equal ratio to the sample, are reduced only by phenolic constituents of the extracts, forming a blue-colored complex. Gallic acid is often utilized as a standard, and the TPC of investigated samples is expressed as gallic acid equivalents (Elkhouly et al. 2020).

Similarly, Total Flavonoid Content (TFC) provides information on the antioxidant capacity of a sample based solely on its flavonoid (a subgroup of phenolic compounds) composition. TFC is assayed using the aluminum chloride colorimetric method, which forms a yellow to yellowish-orange complex, specifically with flavonoids. The intensity of the colored complex formed is proportional to the flavonoid content, and this can be measured as absorbance at a visible light spectrum maximum of 415 nm. To derive a quantitative value from absorbance readings of the TFC assay, extrapolation is performed using a standard curve prepared from either quercetin or catechin (as flavonoid standards). Consequently, TFC results are typically expressed as quercetin or catechin equivalents (Elkhouly et al. 2020).

### Phosphomolybdenum method

The phosphomolybdenum method entails the formation of a green-colored phosphomolybdenum (V) complex, which absorbs maximally at 695 nm, as a result of the reduction of Mo (VI) to Mo (V) by antioxidants (Prieto et al. 1999). This method for estimating the antioxidant capacity of endophytic fungi metabolites closely resembles the protocol used for plant phytochemicals (Hamed et al. 2020). In both plant and endophytic fungi metabolite assays, samples are dissolved in water to a known dilution and mixed with a preparation solution containing ammonium molybdate, sulfuric acid, and sodium phosphate. The mixture is then added to phosphomolybdenum reagent in a 1:1 ratio and incubated at a temperature of 90–100 °C for 60–90 min. During incubation, green-colored reduced Mo(V) complexes are formed, and their intensity, measured with a spectrophotometer, depends on the antioxidant capacity of the metabolites. The Total Antioxidant Capacity (TAC) can be determined by extrapolating the absorbance value from the calibration curve of standard antioxidants such as ascorbic acid or trolox. The antioxidant capacity of the sample is then expressed as milligrams of ascorbic acid equivalents per gram of dry extract (mg AAE/g dry extract) (Nermien et al. 2018). Examples of studies evaluating the antioxidant capacity of endophytic fungi metabolites are summarized in Table 2.

**Table 2:** Some endophytic fungi metabolites and their DPPH radical scavenging activities

Antioxidant Methods	Metabolites	Endophytic fungi	Plant source	Habitat	Plant part	Isolation media/extraction solvent	IC_50_ (µg/mL)	Reference(s)
DPPH	i. Flavipin ii. Epicoccolide A iii. Epicoccolide B iv. Epicocconone v. 3Methoxyepicoccone	*Chaetomium globosum*	*Panax notoginseng*	Terrestrial	Seeds	PDA/Ethyl acetate	i.3.7 ii.5.2 iii.11.6 iv.11.5 v.11.2	(Li et al. 2016b)
	Vitexin	*Dichotomopilus funicola*	Cajanus cajan	Terrestrial	Leaves	PDA/Ethyl acetate	164	(Gu et al. 2018)
	i.trichomerol ii.bisorbicillinolide	*Trichoderma citrinoviride*	*Torreya grandis*	Terrestrial	arils	PDA/Ethyl acetate	i. 38.92 ii. 3.91	(Kong et al. 2023)
	mollicellin O	*Chaetomium* sp.	*Eucalyptus exserta*	Terrestrial	fruits	PDA/Ethyl acetate	71.92	(Ouyang et al. 2018)
	3-(2,6- dihydroxyphenyl)-2-hydroxyacrylic acid	*Penicillium oxalicum*	*Peronema canescens*	Terrestrial	leaves	PDA/Ethyl acetate	31.33	(Elfita et al. 2022)
	i. isovariecolorin I ii.30-Hydroxyechinulin iii. neoechinulin B iv. neoechinulin C v. alkaloid E-7 vi. didehydroechinulin vii. echinulin viii. dehydroechinulin ix. variecolorin H	*Eurotium cristatum* EN-220	*Sargassum thunbergii*	Marine	alga	PDA/Ethyl acetate	i. 20.6 ii.28.5 iii.10.9 iv.12.1 v.10.1 vi.13.3 vii.13.8 viii.6.4 ix.18.7	(Du et al. 2017)
DPPH	i. 2,2′,3,5′-Tetrahydroxy-3′-methylbenzophenone ii. 2,2′,5′-Trihydroxy-3-methoxy-3′-methylbenzophenone iii. 1,4,5-Trihydroxy-2-methylxanthone iv. 1,4,7-Trihydroxy-6-methylxanthone v.8-hydroxyconiothyrinone B vi.4R,8-dihydroxyconiothyrinone B vii.8,11-dihydroxyconiothyrinone B viii.4S,8-dihydroxy-10-Omethyldendryol E ix.4S,8-dihydroxyconiothyrinone B,	*Talaromyces islandicus* EN-501	*Laurencia okamurai*	Marine	alga	PDA/Ethyl acetate	i. 1.26 ii. 1.33 iii. 1.23 iv. 6.92 v. 12 µM vi. 42 vii. 31 viii. 30 ix. 52	(Li et al. 2016a)
	Ascomindones A	*Ascomycota* sp. SK2YWS-L	*Kandelia candel*	Mangrove	leaves	PDA/Ethyl acetate	18.1 µM	(Tan et al. 2016)
	( −)-(7S)-10-hydroxysydonic acid	*Aspergillus* sp. xy02	*Xylocarpus moluccensis*	Mangrove	leaves	PDA/Ethyl acetate	72.1 µM	(Wang et al. 2018)
**Phosphomolybdenum method**	Anofinic acid	***Aspergillus Tubingensis* ASH**	*Hyoscyamus muticus*	Terrestrial	Whole plant	Rice media/Ethyl acetate	111.66–880.66 mg AAE/g dry extract	Elkhouly et al. 2021)
	–	*Fusarium solani and other endophyte species*	*Hibiscus sabdariffa*	Terrestrial	Root, stem, and leaves	PDA/Ethyl acetate and methanol	7.81 ± 0.07 mg/mL Fungal Extracts	(Khalil et al. 2020)
	Novel compounds with unidentified ms/ms data	*Epicoccum nigrum* FAS-14	*Thalassia hemprichii*	Marine	Leaves	Malt Agar and PDA/Ethyl acetate	813.5 mg AAE/g extract	(Hamed et al. 2020)
	i. 1,3- dicaffeoylquinic acid ii. 1,5-dicaffeoylquinic acid	*Alternaria alternata*	*Cynara scolymus* L (artichoke)	Terrestrial	Leaves	PDA and Cellulose/Glucose Czapek	i. 84.8 AAE/g DW ii. 109.8 mg AAE/g DW	(Nermien et al. 2018)
FRAP	isoelemicin (50.8%), terpinen-4-ol (21.5%), eucalyptol (24.3%), oleic acid (19.8%) and β-pinene (10.9%)	*i)Macrophomina phaseolina* *ii)Mycoleptodiscus indicus*	*Gynura procumbens*	Terrestrial	Whole plants	PDA/Ethyl acetate and Methanol	(i) 239.9 mg Fe (II)/g (ii) 44.7 mg Fe (II)/g	(Jamal et al. 2022)
	i. 3-amino-5-(3-hydroxybutan-2-yl)-4-methylphenol (halociline) ii. 1, 3, 6-trihydroxy-7-methoxy-9H-xanthen-9-one (xanthone)	*i)Penicillium citrinum E-346* *ii)Aspergillus flavus RF-03*	*Halocnemum strobilaceum*	Marine	Whole plants	Czapek Yeast Extract Agar/Ethyl acetate	(i)210.895 ± 2.440 μmol/L (ii)58.487 ± 1.345 μmol/L	(Abdel Razek et al. 2020)
	Phenol,2,4-bis(1,1-dimethylethyl)-; Arsenous acid, tris(trimethylsilyl), ester; Silicic acid, diethyl bis(trimethylsilyl) ester and butylated hydroxytoluene	*(i)Grammothele fuligo,* *(ii)Rigidoporus vinctus,* *(iii)****Pichia kudriavzevii***, *(iv)Candida railenensis,* and *(v)Cystobasidium minutum*	*Green algae and Brown algae*	Marine	Whole algae	PDA/Ethyl acetate	(i) 40.88 μg/mL (ii) 39.54 μg/mL (iii) 44.27 μg/mL (iv) 43.36 μg/mL (v) 32.01 μg/mL Ascorbic—21.78 μg/mL	(Harikrishnan et al. 2021)
	Echinulin and neoechinulin A	*(i)Penicillium sect. Exilicaulis* *(ii) Aspergillus chevalieri*	*Halopteris scoparia*	Marine	Whole weed	PDA and Corn Meal Agar (CMA)/Ethyl acetate	(i) 452.8 μg/mL (ii) 3256.1 μg/mL	(Calado et al. 2021)
ORAC	Echinulin and neoechinulin A	*(i)Fusarium incarnatum-equiseti complex* *(ii) Aspergillus chevalieri* *(iii)Nigrospora oryzae* *(iv)Diaporthe sp.*	*Halopteris scoparia*	Marine	Whole weed	PDA and Corn Meal Agar (CMA)/Ethyl acetate	(i) 2579.9 μg/mL (ii) 19,879.6 μg/mL (iii) 2814.1 μg/mL (iv)2823.0 μg/mL	(Vitale et al. 2020) (Calado et al. 2021)
	Fungal Polysaccharide	*Fusarium solani DO7*	*Dendrobium officinale*	Terrestrial	Whole plant	PDA/Ethyl acetate	650 µmol Trolox/g	(Zeng et al. 2019a); (Zeng et al. 2019b)
	adenine alkylresorcinol	*Lasiodiplodia sp*.	*Houttuynia cordata* Thunb	Terrestrial	Whole plant	PDA/Ethyl acetate	4.31 µmol Trolox/g 2.95 µmol Trolox/g 4.25 µmol Trolox/g	(Gao et al. 2016)
ABTS	porphyrin derivatives	Aspergillus species	Tabernaemontana pandacaqui	Terrestrial	Leaves	PDA/Ethyl acetate	866.86 ± 50.0 μM TE/mg extract	(Elhosari et al. 2022)
	i)chaetoglobosin F i)isochaetoglobosin D ii)cytochalasin H	*(i)Chaetomium globosum* WQ *(ii)Phomopsis* sp.	*1)Imperata cylindrica* *ii)Vatica mangachapoi*	Terrestrial	Whole plant	PDA/Ethyl acetate	59 mol trolox/mol	Shen et al. 2014, 2015, 2020)
	resveratrol	*Arcopilus aureus* *Fusarium equiseti*, *Xylaria psidii* *Fusarium solani*	*Vitis vinifera*	Terrestrial	Whole plant	PDA/Ethyl acetate	IC_50_–0.28 μg/ml 0.82 μg/ml, 0.51 μg/ml, and 2.16 μg/ml	(Dwibedi and Saxena 2019)
	Culture filtrate dried powder (CFDP)	*Talaromyces assiutensis*	*Avicennia marina*	Terrestrial	root	PDA/Ethyl acetate	IC_50_–32.01 μg/mL	(Mishra et al. 2021)
	Fungi Extract	*(iii)Pichia kudriavzevii*	*Green algae and Brown algae*	Marine	Whole algae	PDA/Ethyl acetate	IC_50_–38.74 μg/mL	(Harikrishnan et al. 2021)

In a recent study, the endophyte *Aspergillus tubingensis* ASH4 isolated from the *Hyoscyamus muticus* plant was cultured on solid-state media of rice. Metabolites extracted via solvent extraction were analyzed for their Total Antioxidant Capacity (TAC) and Total Phenolic Content (TPC) using the phosphomolybdenum method. A range of 111.66–880.66 mg AAE/g dry extract was obtained for unique extracts from 5 endophytes (Elkhouly et al. 2020). Among these extracts, anofinic acid was isolated and identified for its potent activities, including antimicrobial, anticancer, and antibiofilm properties (Elkhouly et al. 2020). Further interesting studies on the TAC of endophytic fungi are discussed in Table 2.

### 2,2-diphenyl-1-picrylhydrazyl (DPPH) radical scavenging

2,2-Diphenyl-1-picrylhydrazyl (DPPH) is a stable free radical characterized by a deep purple color, which changes to yellow upon neutralization or reduction by electrons (Vitale et al. 2020; Joshua et al. 2021; Okagu et al. 2022; Chukwuma et al. 2023a). This property makes DPPH a suitable reagent widely utilized to evaluate the Total Antioxidant Capacity (TAC) of a metabolite or antioxidant capable of donating an electron. The degree of color change in the presence of an antioxidant can be spectrophotometrically determined at a wavelength of 515 nm, and it is relative to the total antioxidant capacity (Gupta et al. 2020). The method for estimating the TAC of endophytic fungi metabolites using DPPH closely mirrors that used for plants. TAC is quantified through direct extrapolation from a standard curve prepared using standard antioxidants such as ascorbic acid or trolox. Additionally, the percentage scavenging activity of antioxidant metabolites can be estimated from both the absorbance of the sample and the absorbance of the control (which contains only DPPH solution) using the following equation:

1 $$\% Scavanging = \left( {1 - \frac{Absorbance of sample}{{Absorbance of control}}} \right) X 100$$

The DPPH assay has gained popularity in recent studies involving endophytic fungi metabolites/extracts, paralleling trends observed in studies with plant phytochemicals. A summary of selected studies that have adopted the DPPH method for evaluating the antioxidant capacity of endophyte metabolites is provided in Table 2.

For example, using the DPPH method, phenolic compounds obtained from the extract of the fungal endophyte *Cladosporium velox,* isolated from the stem of *Tinospora cordifolia*, exhibited an IC50 value of 22.5 μg/mL, slightly lower than that of ascorbic acid (Singh et al. 2016). Similarly, Khiralla et al. (2015) investigated the antioxidant activity of endophytes from five Sudanese medicinal plants, including *Calotropis procera*, *Euphorbia prostrate*, *Catharanthus roseus, Trigonella foenum-graecum*, and *Vernonia amygdalina*, using the DPPH assay. Among the isolated endophytic fungal strains*, Aspergillus sp*. from *Trigonella foenum-graecum* seeds exhibited the highest IC50 value of 18.0 μg/mL. Additionally, of the eleven endophytic fungi isolated from *Cinnamomum loureiroi* leaves, *Neopestalotiopsis sp.* and Diaporthe sp. demonstrated significant DPPH scavenging activity with IC50 values of 22.92 and 37.61 μg/mL, respectively. Eugenol, lauric acid, myristaldehyde, and caprylic acid were implicated as the primary antioxidants in both fungal extracts (Tanapichatsakul et al. 2019). Further specific illustrations with data showing the antioxidant capacity estimated using the DPPH assay are summarized in Table 2.

### Ferric reducing antioxidant power (FRAP)

The Ferric Reducing Antioxidant Power (FRAP) assay is employed to determine the total antioxidant capacity of a sample (extract or metabolite) based on the reduction of ferric tripyridyltriazine (Fe^3 +—TPTZ) to its ferrous analog (Fe^2 +—TPTZ) in the presence of antioxidants at a low pH (Benzie & Strain 1996). The ferric tripyridyltriazine (Fe^3 +—TPTZ) complex exhibits a stable blue color with maximum absorption at 593 nm, and its blue color intensity diminishes proportionally to the antioxidant capacity of the sample. Thus, a higher reducing power of antioxidants results in a greater reduction of the ferric complex and, consequently, a greater loss of the blue color (Macuphe et al. 2021). The decrease in absorbance values is used to quantitatively extrapolate the total antioxidant capacity (TAC), which is expressed as reducing equivalents of a standard antioxidant (Gupta et al. 2020).

Several studies have adopted the FRAP assay to estimate the TAC of endophytic fungi extracts/metabolites. For instance, in a recent study, two compounds were isolated from the fungal endophytes *Penicillium citrinum E-346* and *Aspergillus flavus RF-03* of *Halocnemum strobilaceum*. Xanthone, isolated from both fungal strains, exhibited a FRAP value of 447.941 ± 37.876 μM equivalent trolox/mL and 1412.400 ± 128.779 μM equivalent ferrous sulfate/mL (Abdel Razek et al. 2020). Similarly, fungal extracts of *Macrophomina phaseolina* and *Mycoleptodiscus indicus* isolated from *Gynura procumbens* were evaluated using the FRAP assay, yielding TAC values of 239.9 and 44.7 mg Fe (II)/g, respectively (Jamal et al. 2022).

In another study, Harikrishnan et al. (2021) utilized the FRAP assay to assess the most promising endophytic fungi extract with optimum TAC among five species isolated from marine macroalgae. *Pichia kudriavzevii* extract exhibited the most promising potential in scavenging ferric iron radicals, with a lower IC50 value of 32.01 μg/mL compared to the other fungi (Harikrishnan et al. 2021). Additionally, two isolates from the seaweed Halopteris scoparia, identified as *Aspergillus chevalieri*, demonstrated remarkable antioxidant capacity, with FRAP values estimated to be 3256.1 and 509.5 μM FeSO4/g of extract (Calado et al. 2021).

Furthermore, the FRAP assay was employed to investigate the effects of culture supplementation on changes in metabolites and TAC. During the extraction of endophytic fungi *Diaporthe fraxini ED2* isolated from *Orthosiphon stamineus*, rosmarinic acid was added as a supplement to the culture media to examine changes in antioxidant capacity. The FRAP assay revealed a higher TAC of 188.41 ± 18.67 µg AAE/mg extract in the supplemented media compared to the control (unsupplemented media), which exhibited lower activity of 53.88 ± 4.31 μg AAE/mg extract (Tan et al. 2022).

### Oxygen radical absorbance capacity (ORAC)

The Oxygen Radical Absorbance Capacity (ORAC) test evaluates the ability of antioxidant compounds to scavenge free radicals using a fluorescent probe and a compound that generates free radicals, particularly peroxyl radicals, which are prevalent in the body (Vitale et al. 2020). In the presence of free radicals, fluorescein undergoes dissociation, leading to a reduction in the fluorescence signal (Tienaho et al. 2020). Antioxidants prevent this dissociation through hydrogen atom transfer, thereby scavenging the free radicals.

Various studies have reported ORAC values for metabolites isolated from endophytic fungi, indicating their antioxidant capacities. For instance, isocoumarins and phthalide from *Colletotrichum sp. CRI535-02*, isolated from *Piper ornatum*, exhibited ORAC units ranging from 1.4 to 14.4, demonstrating varying scavenging capacities (Tianpanich et al. 2011). Flavipin, 1,2-benzenedicarboxaldehyde-3,4,5-trihydroxy-6-methyl, isolated from *Chaetomium globosum* CDW7 from *Gingko biloba* leaf, showed 6.16 ORAC units (Ye et al. 2013).

*Aspergillus chevalieri* isolated from *Halopteris scoparia* exhibited ORAC values of 19,870.6 and 1319.6 μmol TE/g of extract (Calado et al. 2021). Compounds such as corynesidones A and B and two diaryl ethers isolated from *Corynespora cassiicola* L36 from the leaves of *Lindenbergia philippensis* showed ORAC values ranging from 4.3 to 5.9 ORAC units (Chomcheon et al. 2009).

Polysaccharides obtained from endophytic fungi *Fusarium solani* DO7 of the orchid Dendrobium officinale through solid-state fermentation revealed ORAC values of approximately 450 and 650 µmol trolox/g, with antioxidant activities influenced by the presence of monosaccharides (Zeng et al. 2019a, b). Additionally, adenine alkylresorcinol isolated from *Lasiodiplodia sp. i*solated from *Houttuynia cordata Thunb* exhibited potent ORAC compared to epigallocatechin gallate (Gao et al. 2016).

These studies collectively illustrate the diverse antioxidant capacities of metabolites isolated from endophytic fungi, as assessed by the ORAC assay.

### Azino-bis (3-ethylbenzothiazoline-6-sulphonic acid) (ABTS)

The Azino-bis (3-ethylbenzothiazoline-6-sulphonic acid) (ABTS) assay evaluates the ability of antioxidants to scavenge ABTS radicals, which are generated by oxidizing ABTS with a strong oxidizing agent like potassium persulfate, resulting in a deep green solution (Tang et al. 2020). Antioxidants in the sample scavenge these radicals, leading to a loss of green coloration, which can be measured spectrophotometrically. The antioxidant capacity is typically expressed relative to a standard antioxidant like trolox.

Several studies have utilized the ABTS assay to assess the antioxidant activity of metabolites from endophytic fungi. For instance, the ethyl acetate extract of *Aspergillus sp.* isolated from *Tabernaemontana pandacaqui* demonstrated significant ABTS radical scavenging activity, with a Trolox Equivalent Antioxidant Capacity (TEAC) of 866.86 ± 50.0 μM TE/mg extract, attributed to the presence of porphyrin derivatives (Elhosari et al. 2022).

Additionally, cytochalasans derived from *Chaetomium globosum* WQ and *Phomopsis sp.* IFB-E exhibited notable antioxidative properties, with compounds like chaetoglobosin F, isochaetoglobosin D, and cytochalasin H showing significant effects (Shen et al. 2020). *Arcopilus aureus*, isolated from *Vitis vinifera,* displayed considerable ABTS radical scavenging activity, with an IC50 value of 0.28 mg/mL (Dwibedi & Saxena 2019).

*Talaromyces assiutensis* isolated from *Avicennia marina* roots showed an IC50 of 32.01 mg/mL against ABTS radical (Mishra et al. 2021). *Pichia kudriavzevii* from a marine alga and *Colletotrichum alatae LCS1* from *Lycopodium clavatum* demonstrated IC50 values of 38.74 μg/mL and 23.38 μg/mL, respectively, for ABTS radical scavenging activity (Harikrishnan et al. 2021; Santra & Banerjee 2022).

Moreover, *Diaporthe fraxini* ED2 supplemented with rosmarinic acid exhibited enhanced ABTS radical scavenging activity, with an IC50 of 17.83 µg/mL compared to unsupplemented broth (Tan et al. 2022). Compounds isolated from *Talaromyces islandicus* EN-501 showed ABTS radical scavenging activities ranging from 0.58 to 2.35 µg/mL, surpassing that of ascorbic acid (Li et al. 2016b). *Pestalotiopsis mangiferae* isolate HL14 from *Bruguiera sexangular* yielded a compound with potent ABTS radical scavenging activity, with an IC50 value of 0.49 mg/mL (Zhang et al. 2021).

These findings underscore the diverse and significant antioxidant capacities of metabolites derived from endophytic fungi, as evaluated by the ABTS assay.

### Other methods

Endophytic fungi, residing symbiotically within plant tissues, have emerged as promising sources of bioactive compounds with diverse pharmacological properties, including antioxidant activities. Understanding the mechanisms underlying the antioxidant activities of endophytic fungi is crucial for harnessing their therapeutic potential. While several methods exist for assessing antioxidant capacity, some are seldomly employed for evaluating endophytic fungi including hydrogen peroxide scavenging assay, nitric oxide radical scavenging assay, and superoxide radical scavenging assays.

The endophytic isolate *Arcopilus aureus*, derived from *Vitis vinifera*, demonstrated remarkable antioxidant activity, with IC_50_ values of 0.12 mg/mL for metal ion chelating and hydrogen peroxide scavenging assays, as well as 0.08 mg/mL for the nitric oxide radical scavenging assay (Dwibedi & Saxena 2019). Additionally, a demethylated derivative of fusarentin 6-methyl ether and monocerin from *Colletotrichum sp.,* isolated from *Piper ornatum*, exhibited significant superoxide radical scavenging activity, with IC50 values of 4.3 and 52.6 μM, respectively (Tianpanich et al. 2011).

Among the six flavonoid-producing endophytes isolated from *Conyza blinii* H. Lé leaves, two demonstrated potent scavenging activity against hydroxyl radicals, with IC_50_ values of 0.19 mg/mL, surpassing that of ascorbic acid with an IC50 value of 0.96 mg/mL (Tang et al. 2020). Furthermore, crude fungal extracts from *Hibiscus sabdariffa* displayed significant hydrogen peroxide scavenging abilities, ranging from 93 to 99% (Khalil et al. 2020).

Three dibenzo-α-pyrones, namely, 3-epi-dihydroaltenuene A, 4-hydroxyalternariol-9-methyl ether, and altenuisol, isolated from Alternaria sp. Samif01, an endophyte from *Salvia miltiorrhiza* Bunge roots, exhibited notable hydroxyl radical scavenging activities, with EC_50_ values of 267.1, 68.3, and 261.5 µM, respectively (Tian et al. 2017). Similarly, Ascomindones A − C, isolated from *Ascomycota sp.* SK2YWS-L from the marine mangrove plant *Kandelia candel*, demonstrated strong hydroxyl radical scavenging activities, with IC_50_ values ranging from 80 to 100 μM (Tan et al. 2016).

Additionally, the fungal extract from *Colletotrichum alatae* LCS1, obtained from *Lycopodium clavatum*, exhibited significant H_2_O_2_ scavenging ability, with an IC_50_ value of 52.75 µg/mL (Santra & Banerjee 2022).

## Limitations and prospects for future studies

Despite the plethora of antioxidant metabolites provided by endophytic fungi, their exploration remains incomplete due to various constraints. Firstly, the elaborate procedures involved in isolating, culturing, purifying, and characterizing endophytes have led many studies to prioritize process optimization over elucidating the comprehensive biochemical properties of these metabolites. This limitation underscores the need to shift scientific focus toward uncovering the unexplored activities of endophytic fungi, both for bioprocesses and industrial applications. Moreover, researchers aiming to delve deeper into these untapped potentials often encounter challenges related to the poor stability and low yield of metabolites from endophytic fungi, hindering their ability to meet industrial standards (Wang et al. 2023).

In recent years, a few scholars have begun investigating methods to enhance the stability and industrial viability of antioxidant metabolites derived from endophytic fungi. One prominent approach involves the application of nanotechnologies to formulate these metabolites into nanostructures, which has demonstrated improvements in stability and bioactivity, including total antioxidant capacity (Gezaf et al. 2022). Various nanoparticles, such as silver nanoparticles, nano-iron oxides, zinc oxide, and gold nanoparticles, have been explored for this purpose (Ganesan et al. 2020; Ameen et al. 2023). Nanosystems offer the potential for enhanced stability, efficient delivery, amplified activities, and potentially reduced toxicity (Rana et al. 2020).

Another promising avenue that warrants further exploration is the use of genetic tools to upregulate desirable metabolites in endophytic fungi. Given their unicellular or micro-multicellular nature, endophytic fungi are amenable to genetic manipulation and can be subjected to induction mechanisms, utilizing appropriate inducers to promote overproduction (Parra-Rivero et al. 2020). Improved purification can be achieved by incorporating affinity tags such as polyhistidine tags (6xHis tag) during cloning.

Furthermore, while endophytic fungi often exist in symbiotic relationships with plants, they may pose risks to human health. Therefore, it is crucial to investigate the antigenic potential of these metabolites to ensure biosafety and evaluate potential toxicity. Exploring the possibility of administering endophytic fungi extracts as homogenates, akin to single-cell protein, presents an intriguing avenue for future research. This entails examining their toxicity levels and assessing whether administering them as homogenate extracts, similar to herbal mixtures, enhances antioxidant capacity or other bioactivities.

Overall, addressing these limitations and exploring these avenues holds significant promise for advancing our understanding and harnessing the potential of endophytic fungi metabolites for various applications.

## Conclusion

Endophytic fungi, ubiquitous symbionts found in both medicinal and non-medicinal plants, have emerged as promising sources of secondary metabolites with potent antioxidant capacities, holding significant potential for health and biological applications. However, despite their recognized importance, endophytic fungi remain largely underexplored to date. The pervasive issue of oxidative stress, implicated in various metabolic dysfunctions and disease pathogenesis, has spurred the quest for novel antioxidant and radical scavenging compounds. In this context, endophytic fungi have garnered attention as a reservoir of such metabolites.

This study has provided an overview of the methods employed for the isolation, characterization, and estimation of total antioxidant capacity (TAC) of endophytic fungi-derived metabolites. Despite the burgeoning interest in this field, several limitations have been identified, underscoring the need for further investigation. Key among these limitations are the challenges associated with the isolation, purification, and characterization of endophytic metabolites, as well as concerns regarding their stability and industrial applicability.

Looking ahead, there are promising prospects for future research endeavors in this area. Strategies such as the application of nanotechnologies for enhancing stability and bioactivity, genetic manipulation to upregulate desirable metabolites, and exploration of the antigenic potential and toxicity profiles of endophytic metabolites present exciting avenues for exploration. By addressing these limitations and delving into these prospects, researchers can unlock the full potential of endophytic fungi metabolites for various biomedical and industrial applications.

In summary, while much progress has been made in uncovering the antioxidant potential of endophytic fungi, there remains a wealth of untapped opportunities awaiting exploration. Continued research efforts in this field hold the promise of yielding novel insights and applications that could significantly impact human health and well-being.

## Data Availability

All data generated or analyzed during this study are included in this published article.
